# App Usage Factor: A Simple Metric to Compare the Population Impact of Mobile Medical Apps

**DOI:** 10.2196/jmir.4284

**Published:** 2015-08-19

**Authors:** Thomas Lorchan Lewis, Jeremy C Wyatt

**Affiliations:** ^1^ Kingston Hospital National Health Service (NHS) Trust London United Kingdom; ^2^ Leeds Institute of Health Sciences University of Leeds Leeds United Kingdom

**Keywords:** mHealth, medical app, mobile phone, metric, risk assessment, medical informatics apps, population impact, mobile health, patient safety, mobile app

## Abstract

**Background:**

One factor when assessing the quality of mobile apps is quantifying the impact of a given app on a population. There is currently no metric which can be used to compare the population impact of a mobile app across different health care disciplines.

**Objective:**

The objective of this study is to create a novel metric to characterize the impact of a mobile app on a population.

**Methods:**

We developed the simple novel metric, app usage factor (AUF), defined as the logarithm of the product of the number of active users of a mobile app with the median number of daily uses of the app. The behavior of this metric was modeled using simulated modeling in Python, a general-purpose programming language. Three simulations were conducted to explore the temporal and numerical stability of our metric and a simulated app ecosystem model using a simulated dataset of 20,000 apps.

**Results:**

Simulations confirmed the metric was stable between predicted usage limits and remained stable at extremes of these limits. Analysis of a simulated dataset of 20,000 apps calculated an average value for the app usage factor of 4.90 (SD 0.78). A temporal simulation showed that the metric remained stable over time and suitable limits for its use were identified.

**Conclusions:**

A key component when assessing app risk and potential harm is understanding the potential population impact of each mobile app. Our metric has many potential uses for a wide range of stakeholders in the app ecosystem, including users, regulators, developers, and health care professionals. Furthermore, this metric forms part of the overall estimate of risk and potential for harm or benefit posed by a mobile medical app. We identify the merits and limitations of this metric, as well as potential avenues for future validation and research.

## Introduction

### Overview

The growth in popularity of health and medical apps for health care professionals and patients is widely recognized given their numerous successful uses in a number of health care domains, including clinical health care delivery, education, and health promotion [1-4]. However, a number of concerns regarding the reliability and accuracy of apps have arisen, leading to calls for some form of quality assessment [5-7].

Evaluating the quality of mobile apps is a notoriously difficult problem which currently has no standard solution. Ideally, every medical app should be evaluated and tested by a range of experts to ensure its suitability and applicability to medicine. In practice, this is impossible to achieve given the exponential growth in the app market, low barriers to entry, limited resources, and rapid pace of development [6]. A number of models have been proposed to help clinicians, app developers, regulators, and commissioning bodies to assess the quality of mobile apps, although it remains to be seen which model has the most utility in practical terms [5,8,9].

A previous paper proposing a framework for risk assessment for mobile medical apps identified many components that increase the potential for harm [5]. The components included inherent factors to the app, such as functionality, content, complexity, and lack of a fail-safe. External factors included the app user, inappropriate usage, inadequate training, and the likelihood of an error being detected. Furthermore, this paper suggested that an important component of the potential harm caused by a medical app is the overall impact that a mobile app has on a given population [5]. Lewis et al noted, "Risk is proportional to the number of patients affected, so disease prevalence or similar indices of the number of people likely to be affected by an error need to be considered."

It follows that a less harmful app used by a large population could pose a greater overall population safety risk than a more harmful app used by a small population. Therefore, it is clear that there is a need to develop a metric that will assess the population impact of mobile medical apps and will allow subsequent comparison across different disciplines.

### The Problem: Assessing the Impact of a Specific App

There are a limited number of options currently available to assess the impact of a mobile medical app on any given population; these are shown in Table 1 and are ranked in order of accuracy.

It is important for the various stakeholders to be able to estimate and compare the likely population impacts of specific apps for the reasons shown in Table 2.

It is critical to be able to assess the number of people at risk from an unsafe app at any given time and currently there is no clear method of assessing this. Our objective was to propose, develop, and model a simple metric that can be used to estimate and compare the likely impact of a specific app on a population.

**Table 1:** Models currently in use for assessing the impact of a mobile app on a given population.

Assessment tool^a^	Examples	Advantages	Disadvantages
Detailed app analytics	High level metrics such as active users, time spent on app, and ethnographic and epidemiological data	Gold standard in terms of data detail Would enable precise population impact to be measured	Large volumes of data Not currently practical Relies on app developers releasing crucial business information Developer bias
mHealth studies [10]	Numerous mHealth studies testing the validity of mobile apps for health care	Rigorous independent trials Often have detailed metrics available Often note the quality of an app	Often focus on one specific app Not many studies available Not easy to compare apps from different disciplines
Number of app downloads	Basic metric available from a number of sources	Can easily compare apps from different disciplines	Information not easily accessible Many users only download an app for trial purposes No information about how often an app is used No information about intended audience Often not reported accurately
Educated guesswork	N/A^b^	Minimal knowledge required to provide estimate	Not accurate or precise Wide observer bias

^a^Assessment tools are ranked in order of accuracy.

^b^Not applicable (N/A).

**Table 2:** Key reasons for use of population impacts of mobile apps by stakeholders.

Stakeholder	Reason for estimating app impact on population
Regulator	To estimate and compare the overall risks posed if the app is unsafe, and to decide on the appropriate regulatory measures
Guideline developer (eg, NICE^a^)	To understand the potential for population benefit from effective apps To help understand the impact of an app from a public health perspective
App developer	To justify investment decisions To guide update strategy
App users	May use the population impact as a surrogate indicator for quality
Clinicians advising users about the app	May use the population impact as a surrogate indicator for quality
Health insurers and funding schemes	To understand the likely payback from approving reimbursement of the cost of the app
Health economists	As part of an estimate of cost effectiveness of the app
App stores	Could utilize AUF^b^as part of their ranking algorithm Surrogate marker for quality

^a^National Institute for Health and Care Excellence (NICE).

^b^App usage factor (AUF).

## Methods

### Identification of a Simple Metric: App Usage Factor

A broad literature search for existing metric systems in use was carried out. The two authors of this paper (TLL, JCW) searched for relevant papers with regard to their suitability for use when applied to medical apps, however, none were found. The search was expanded to look for metrics in use in other aspects of technology. We felt that there were some useful analogies outside health care; these included *passenger miles* for comparing CO_2_emissions from alternative transport methods, the *readership* of newspapers and magazines (number of sales × number of readers per paper), and common *website metrics*, which capture the number of unique visitors × time spent per visit.

We also brainstormed the criteria for a useful metric. In our view, a good metric should display the following characteristics:

1. Simple to calculate from readily available information

2. Reflects both the number of users and the frequency of use

3. Generates a single, understandable figure within the range of 0 to 10, despite the hugely varying number of users per app (at least a million-to-one ratio)

4. Relatively stable over time for each app

5. Can be used as a denominator for adverse incident reports

6. Makes intuitive sense to users in the same way as does the Richter scale, for example.

7. Has good interobserver agreement and reliability.

Our proposed metric, the app usage factor (AUF), is defined as the logarithm of the product of the total number of active users of a mobile app (*A*_*U*_) with the median number of daily uses of the aforementioned app (*D*_*U*_). The formula for calculating the metric can be seen in equation 1 as follows:

AUF=log_10_(*A*_*U*_×*D*_*U*_) (1)

There are a number of points worth considering that enable this metric to fulfil all the desired characteristics. Certain considerations for the measurement of *A*_*U*_and *D*_*U*_include geographic boundaries (eg, AUF could be global or country specific), operating system version (eg, AUF could differ for iPhone and Android platforms), and app version number, which would need to be specified or standardized in order to make a meaningful assessment of the AUF.

There are also specific temporal considerations when calculating *A*_*U*_ *, D*_*u*_, and AUF, in particular, the following:

1. The figure for each variable should be assessed as "stable," for instance, not changed significantly over the past 30 days. This is because the use of apps by users can change extremely rapidly, for example, due to media hype surrounding the release of a new app.

2. The AUF for a specific app can change over time as the app ecosystem evolves. Specific issues that may be encountered include app or operating system updates, which may significantly affect the functionality of the app.

On the basis of the considerations above, it is reasonable to calculate the AUF for a specific app on a quarterly basis, at minimum, or 30 days following a major app update. Given the well-recognized speed of app updates, this may well require further refinement [11].

### Validation of the App Usage Factor Metric Against Proposed Criteria

#### Overview

Any novel metric requires appropriate validation to ensure its suitability and accuracy for the proposed task. A series of computer simulation models were constructed that allowed specific app usage scenarios to be explored. The objective was to validate the AUF metric against the desirable metric criteria, thus confirming its suitability and applicability for practical use. Three specific scenarios were modelled using Python [12], a high-level, general-purpose programming language, as follows:

1. Exploring stability of AUF as a function of *A*_*u*_and *D*_*u*_, including determination of metric limits

2. Simulated app ecosystem model

3. Temporal stability of AUF*.*

#### Exploring Stability of the App Usage Factor as a Function of Auand Du, Including Determination of Metric Limits

A 200×200 linear spaced grid was constructed in Python to simulate the behavior of the metric with calculated values of the AUF based on 0<*A*_*U*_<100,000 and 0<*D*_*U*_<50. A secondary iterative process was used to explore the limits of the AUF while still returning usable results. Specific positive and negative limits were identified.

#### Simulated App Ecosystem Model

Figures from a recent study suggest that there are now 20,000 health and medical apps available to download from the major online app stores [7]. In order to validate the new metric, Python was used to construct a simulated dataset with 20,000 "apps," each with a random number of daily "uses" and a random number of active "users." The values for *A*_*U*_and *D*_*U*_were randomly distributed using a negative exponential probability distribution; this is a continuous probability distribution which describes the time between events in a Poisson process (ie, a process in which events occur continuously and independently at a constant average rate [13]). This probability function was chosen to reflect the decreasing probability of an app being *both* widely used (*A*_*U*_) *and* used multiple times per day (*D*_*U*_). The probability distribution function for an exponential distribution is shown in equation 2.

*P*(*x*)=*λe* ^-λx^(2)

An iterative process was used to identify values for lambda in order to identify suitable limits for the maximum number of daily activities, and the maximum number of active users [14]. This was determined to be 0.00001 and 0.4 for *A*_*U*_and *D*_*U*_, giving maximum numbers of 1 million users and 30 uses per day, respectively.

#### Temporal Stability of the App Usage Factor

In order to assess the temporal stability of the AUF, the behavior of a single app was modelled as a function of time. The strength of the logarithmic component of the AUF is its ability to act as a damping system to external ecosystem factors (eg, media hype). The aim was to show that the AUF would not change dramatically in response to these factors. In order to test this, a series of external ecosystem factors would be applied to the simulation to observe how the AUF changed. Key criteria for the simulation were as follows:

1. *D*_*U*_calculated to be a random float value between minimum and maximum values of *D*_*U*_(*D*_*U MIN*_and *D*_*U MAX*_, respectively) according to a uniform probability distribution each day

2. *A*_*U*_calculated to increase/decrease by x users each day, where x is a float value determined by a uniform probability distribution between minimum and maximum values of *A*_*U*_(*A*_*U MIN*_and *A*_*U MAX*_, respectively)

3. AUF calculated according to values of *A*_*U*_and *D*_*U*_as described by the metric and modelled as a function of time for a period of 2 years.

Specific functions were applied to the model, which intended to simulate the following external app ecosystem events: initial market launch, positive media publicity, negative media publicity, and app/operating system updates. The AUF for the simulated app was then plotted as a function of time to analyze temporal behavior. The values for initial *A*_*U*_(*A*_*U INITIAL*_), *A*_*U MIN*_, *A*_*U MAX*_, *D*_U MIN,_and *D*_*U MAX*_for each external ecosystem event are shown below in Table 3.

**Table 3:** Initial data used to model the characteristics of the AUF as a function of time for a single mobile app.

External ecosystem event	Day number	*A*_*U MIN*_^a^	*A*_*U MAX*_^b^	*D*_*U MIN*_^*c*^-*D*_*U MAX*_^d^
Initial market launch	1^e^(No. of users initially set at 50)	-50	50	10-20
Daily market fluctuation	All days other than those below	-50	50	10-20
Positive media publicity	100-110	50	500	10-20
Negative media publicity	350-360	-500	50	10-20
App version/operating system update	501^f^(No. of users reset to 500)	-50	50	10-20
Users upgrade to latest version	500-650	-20	250	10-20

^a^Range for minimum number of active users of a mobile app (*A*_*U MIN*_).

^b^Range for maximum number of active users of a mobile app (*A*_*U MAX*_).

^c^Range for minimum median number of daily uses of an app (*D*_*U MIN*_).

^d^Range for maximum median number of daily uses of an app (*D*_*U MAX*_).

^e^Initial number of active users of a mobile app (*A*_*U INITIAL*_) on day 1 (initial market launch)=50.

^f^At day 501, the number of active users was reset to 500 to simulate app version/operating system update.

## Results

### Stability of the App Usage Factor as a Function of AUand DU

**Figure 1:**
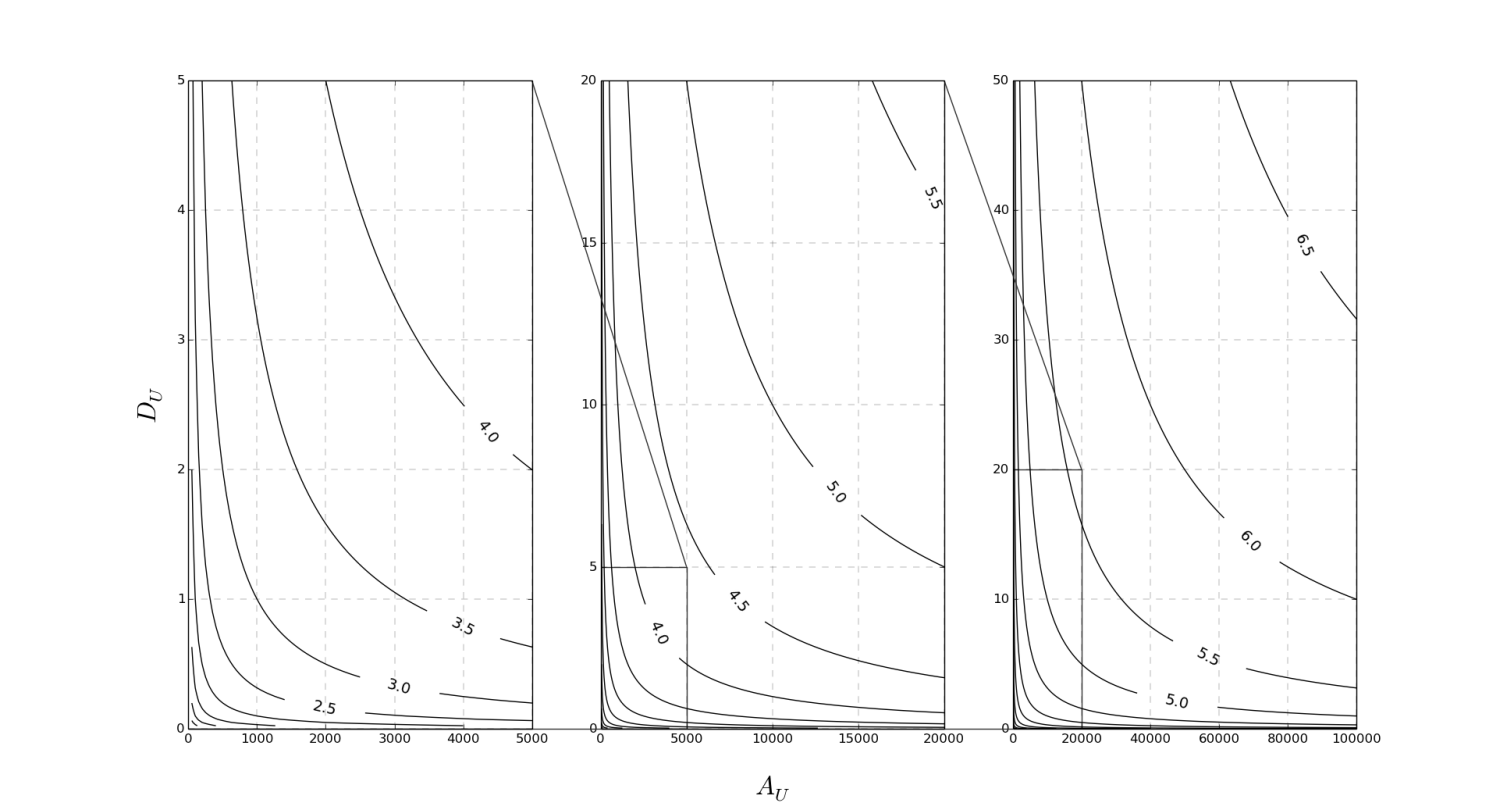
A contour plot illustrating the stability of the app usage factor as a function of Au and Du, including determination of metric limits.

The results of our model shown in Figure 1 highlight the value for AUF (contour lines) as a function of *A*_*U*_and *D*_*U*_. The metric remains stable (ie, AUF > 0) provided *A*_*U*_× *D*_*U*_is greater than 1. It is reasonable to assume that if *A*_*U*_× *D*_*U*_were less than 1, then the specific app would be rarely used, if at all. In practical terms, this relates to a scenario where an app is used regularly once a month by 50 people or less, which was deemed to be an acceptable minimum standard.

### Simulated App Ecosystem Model

**Figure 2:**
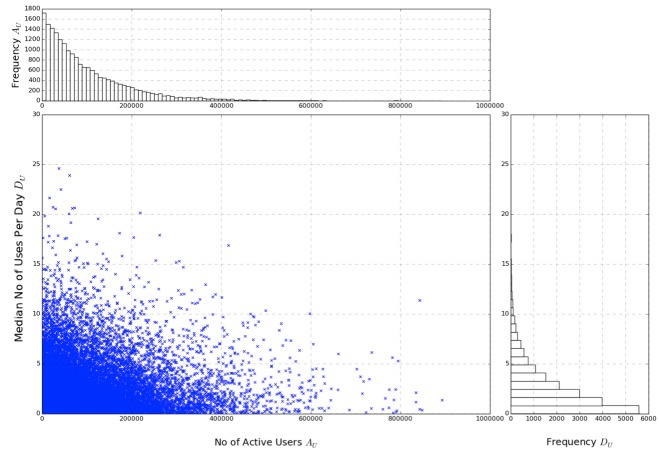
A combined scatterplot (input data, left) and histogram (relative frequency of both Au and Du, right) showing the initial sample dataset of 20,000 mobile medical apps.

**Figure 3:**
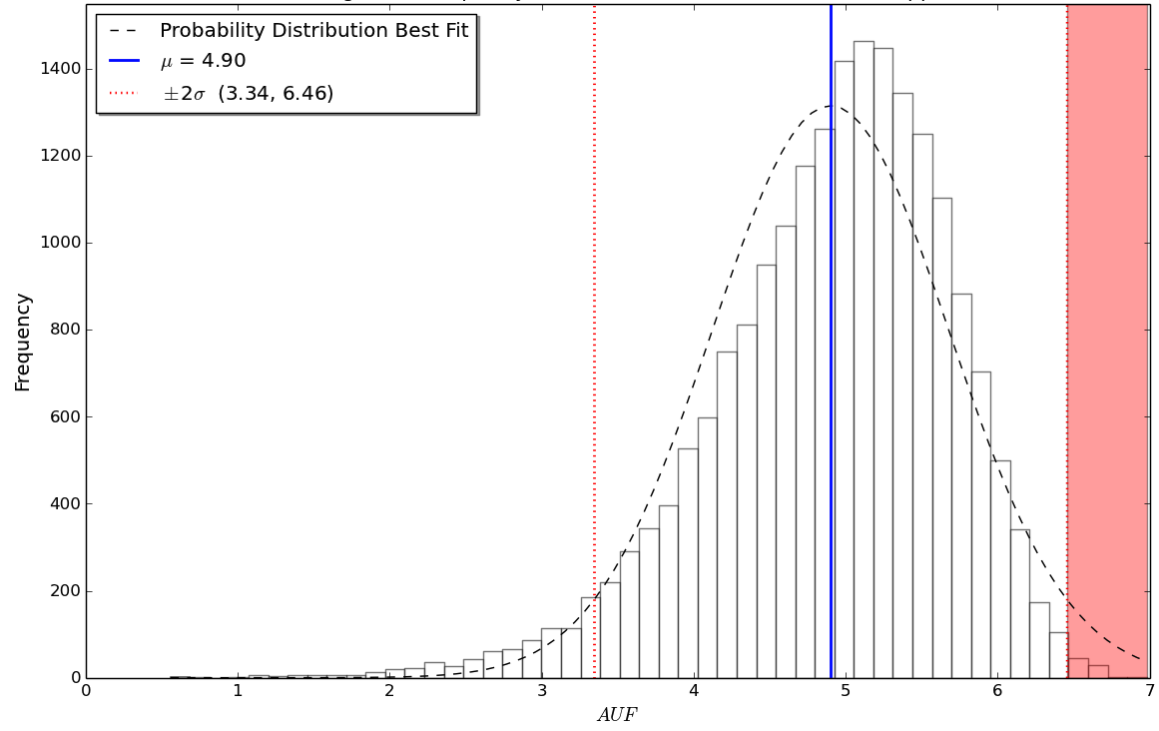
A histogram showing the frequency distribution of the app usage factor for the sample dataset of 20,000 simulated mobile medical apps, including mean and standard deviation for the data.

The input data can be seen on the left in Figure 2, with the relative frequency of both *A*_*U*_and *D*_*U*_plotted as histograms on the right. Each data point represents an individual mobile app with an independent, randomly assigned *A*_*U*_and *D*_*U*_. The AUF was then calculated and plotted as a histogram against frequency as shown in Figure 3. As a result of the logarithmic scaling factor, each unit increase in AUF represents a factor of 10 for impact on the population, similar to the Richter scale.

Apps with a similar AUF can be considered to have a comparable population impact to each other, while simultaneously giving a useful indication of the scale of users affected (see Table 4). Distribution of the sample results can be seen in Figure 3. The interquartile range for AUF was calculated to be 4.45 to 5.45. The mean AUF was 4.90, with the standard deviation calculated to be 0.78.

### Temporal Stability of the App Usage Factor

**Figure 4:**
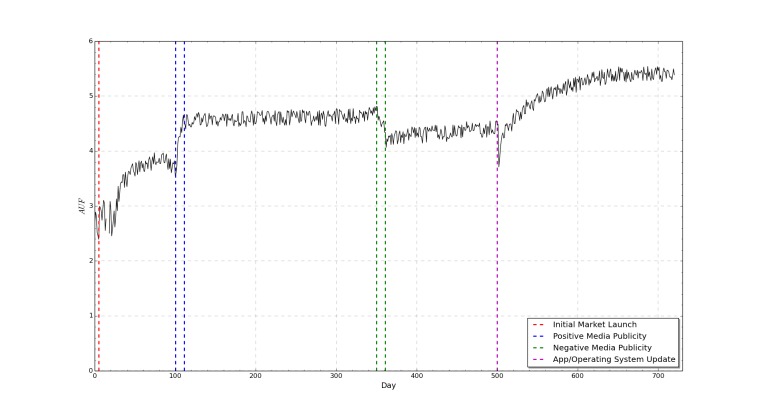
A graph showing app usage factor as a function of time for a single mobile app which is subject to a number of simulated app ecosystem events.

The temporal simulation shown in Figure 4 shows that the AUF will tend toward a relatively stable state despite market perturbations. Minor daily fluctuations in *A*_*U*_and *D*_*U*_are effectively dampened by the addition of the logarithmic factor. Temporal simulations carried out without the logarithmic factor show an increased sensitivity to small changes in *A*_*U*_and *D*_*U*_, which lead to an overall decrease in stability over time. Our simulations suggest that there is a lag time present between a major app ecosystem perturbation and the corresponding change in AUF. This is likely to represent the time taken for information to reach the affected user base and is therefore dependent on the number of users and the magnitude of the market perturbation. It follows that a small market perturbation affecting a small number of users is unlikely to significantly affect the AUF*.* On the basis of this simulation, it is appropriate to delay measuring AUF for 30 days after any minor market perturbation and 80 to 100 days after a large market perturbation (eg, app launch, operating system update).

## Discussion

### Merits and Limitations of the App Usage Factor Metric

Use of the AUF as a metric to assess the population impact of a mobile medical app has many potential benefits for health care professionals, developers, and regulators alike. People who use this metric should be able to make a comparison of the AUF with real-world usage of a particular app, as can be seen in Table 4.

The biggest limitation of this metric is obtaining the two key pieces of information: How many active users there are (*A*_*U*_) and how many times a day the app is used (*D*_*U*_). It is not possible to accurately calculate the AUF without approximate figures for these variables. Identification of *A*_*U*_and *D*_*U*_could be facilitated through the following means:

1. Voluntary release by app developers/app store companies to an independent body where possible, perhaps as part of a self-certification process. The AUF could be calculated by developers and release of this data does not in itself release confidential information.

2. Compulsory release as part of a formal regulatory process, for example, to gain Food and Drug Administration (FDA) regulation approval.

3. Survey of a target population with subsequent data extraction and extrapolation of log files associated with app usage.

There are a number of further considerations of the app usage factor for risk assessment of mobile apps, particularly when utilizing AUF to estimate population impact of an app.

Risk-based regulatory models such as those utilized by the FDA [15] and the Medicines and Healthcare products Regulatory Agency (MHRA) [16] could potentially target apps with a high AUF (ie, a large population impact) purely on the basis of potential negative impact to a population, as suggested in the red highlighted area in Figure 3. Previous risk assessment analysis of mobile apps identified that approximately 0.5% of apps require this formal regulatory assessment. In our opinion, it is therefore reasonable to identify the apps which have the highest population impact and assess their safety on a case-by-case basis. Identification of these apps is accomplished by identifying all apps that fall more than 2 standard deviations from the mean AUF. In our sample data, this equated to 82 out of the 20,000 simulated apps, a feasible proportion of 0.41% of the apps which could require assessment pending formal regulation; these are highlighted in red in Figure 3. In our simulation, this equates to an app with an AUF greater than 6.46.

A limitation of the AUF in estimating the potential risk of a mobile app is when the number of active users is high within a potentially small user base. This is illustrated by the following thought experiment. A potentially harmful app used regularly once a day by 600 users out of a population of 800 users has a higher chance of causing harm to this user group than the AUF of 2.8 for this scenario alone would suggest. This thought experiment confirms that the AUF is a measure of *population* impact and there are several contextual and other factors that contribute to overall risk posed by a medical app [2].

**Table 4:** Equivalent population impact of an app based on its corresponding AUF.

App usage factor (AUF)	Equivalent active user daily actions (*A*_*U*_^a^× *D*_*U*_^b^)
6	1,000,000
5	100,000
4	10,000
3	1000
2	100

^a^Number of active users of a mobile app (*A*_*U*_).

^b^Median number of daily uses of an app (*D*_*U*_).

### Conclusion

A key component when assessing app risk and potential harm is understanding the potential population impact of each mobile app. Our new metric would have many potential uses for a wide range of stakeholders in the app ecosystem, including users, regulators, developers, and health care professionals. Furthermore, this metric forms part of the overall estimate of risk and potential for harm or benefit posed by a mobile medical app [2]. We developed and explored the characteristics of a novel but simple, easily calculated metric to assess the likely population impact of a medical app using a sample database of 20,000 apps modelled using a computer simulation. This modelling showed that our proposed metric, AUF, remained stable over time and at extremes of user numbers and daily usage rates, thereby confirming its suitability for further testing in a health care context. We are confident that using this metric will help the population impact of a specific app to be estimated and compared with similar apps. It is important to note that AUF forms but one component of the overall risk and harm potential posed by a specific app. Users should take the AUF into consideration alongside inherent and external risk factors when deciding whether to use an app in clinical practice. For now, the next stage in the validation process is to calculate the app usage factor for a number of health and medical apps using actual usage and population data.

## Abbreviations

AU: number of active users of a mobile app
AUINITIAL: initial AU
AU MAX: maximum value of AU
AU MIN: minimum value of AU
AUF: app usage factor
DU: median number of daily uses of an app
DU MAX: maximum value of DU
DU MIN: minimum value of DU
FDA: Food and Drug Administration
MHRA: Medicines and Healthcare products Regulatory Agency
MRC: Medical Research Council
N/A: not applicable
NICE: National Institute for Health and Care Excellence
